# Mixed methods

**DOI:** 10.1371/journal.pgen.1008950

**Published:** 2020-07-15

**Authors:** David J. Balding, Gregory S. Barsh, Gregory P. Copenhaver, Chengqi Yi

**Affiliations:** 1 Melbourne Integrative Genomics, School of BioSciences and School of Mathematics & Statistics, University of Melbourne, Parkville, Victoria, Australia; 2 HudsonAlpha Institute for Biotechnology, Huntsville, AL, Department of Genetics, Stanford University School of Medicine, Stanford, California, United States of America; 3 Department of Biology and the Integrative Program for Biological and Genome Sciences, University of North Carolina at Chapel Hill, Chapel Hill, North Carolina, United States of America; 4 State Key Laboratory of Protein and Plant Gene Research, School of Life Sciences, Peking University, Beijing, China

**Fig 1:**
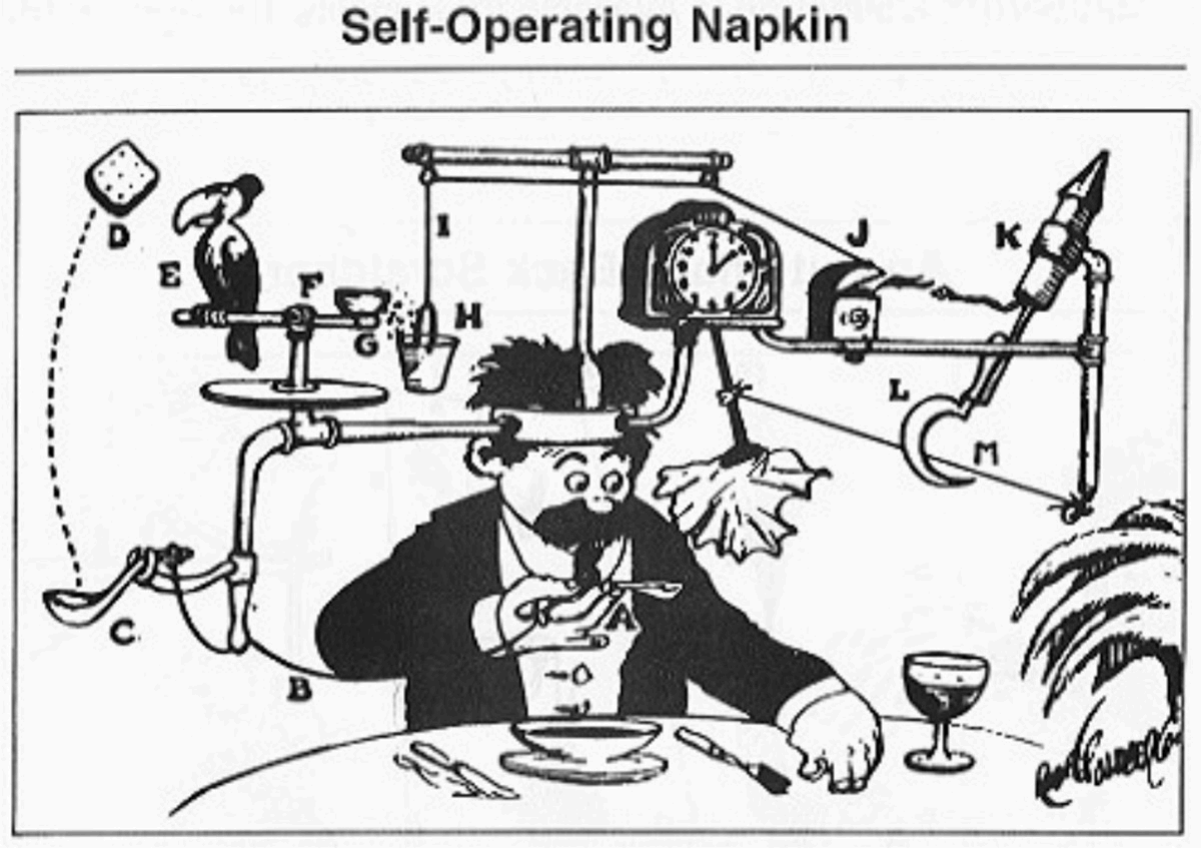
Rube Goldberg’s “Self-Operating Napkin”. Rube Goldberg, Wikimedia Commons, CC0.

Why does this 1931 image (Fig 1), and many more like it by the Pulitzer Prize winning cartoonist Rube Goldberg, have such enduring appeal? Because they capture two things near and dear to our hearts: creativity and problem solving, albeit in a singularly endearing and comical manner. These two elements are also what drive us as scientists to develop new methods—though hopefully more efficient than the one shown—and methods in turn provide us the vital tools we use to propel the scientific enterprise forward. Inspired by this virtuous cycle of scientists creating methods to facilitate more and better science, we are excited to announce that PLOS Genetics is launching a new Methods Section for the journal, led by its founding Section Editors Drs. Balding and Yi.

Evaluating and publishing methods papers presents a challenge because they are as varied as the components of a Goldbergian design. On one end of the spectrum are purely analytical methods—often blending statistical and computational approaches—developed to apply new ways of thinking about or analyzing a question or dataset. Think base-calling or imputation. On the other end of the spectrum are experimental methods developed to apply new principles or discoveries to physical things, from molecules to whole organisms. Think ChIP-seq or enzyme-based gene editing. While colloquially referred to as “dry bench” and “wet bench”, these ends of the spectrum seek a common goal—to provide tools that help us learn more about the living world around us. More important, the approaches are often interdependent, e.g. new technologies create needs and opportunities for new methods to interpret the data. Our editorial team is purposefully designed to capture both ends and embrace all the creative combinations that exist in between.

Given such a broad remit, what criteria will we use to evaluate Methods manuscripts? As with all of our articles we will be guided by the extent to which the methods will be of interest to the broad readership of the genetics community—those that transcend particular model systems or narrow categories of data will be of greatest interest. Methods, like any aspect of science, build on prior work, but we will be most excited about those that represent a substantial leap forward rather than incremental progress. Methods that represent a novel application of an existing approach, or only minor changes to it, won’t be accepted for the Methods section. Finally, while it is more difficult to gauge, methods that have the potential to serve as long-lasting standards, rather than be rapidly displaced, will be prioritized.

In creating the Methods section, we take heed of the words of Sydney Brenner, “Progress [in science] depends on the interplay of techniques, discoveries, and new ideas, probably in that order of decreasing importance” [1]. We seek to recognize and publish techniques and approaches that will lead to new discoveries and new ways of thinking about biology. We hope that you are as excited as we are by the launch of our new Methods Section and we look forward to seeing the products of your creativity submitted to the journal.
